# S1P_2_ contributes to microglial activation and M1 polarization following cerebral ischemia through ERK1/2 and JNK

**DOI:** 10.1038/s41598-019-48609-z

**Published:** 2019-08-20

**Authors:** Arjun Sapkota, Bhakta Prasad Gaire, Min-Gu Kang, Ji Woong Choi

**Affiliations:** College of Pharmacy, Gachon University, 191 Hambakmoero, Yeonsu-gu, Incheon 21936 Republic of Korea

**Keywords:** Stroke, Microglia

## Abstract

Sphingosine 1-phosphate (S1P) signaling has emerged as a drug target in cerebral ischemia. Among S1P receptors, S1P_2_ was recently identified to mediate ischemic brain injury. But, pathogenic mechanisms are not fully identified, particularly in view of microglial activation, a core pathogenesis in cerebral ischemia. Here, we addressed whether microglial activation is the pathogenesis of S1P_2_-mediated brain injury in mice challenged with transient middle cerebral artery occlusion (tMCAO). To suppress S1P_2_ activity, its specific antagonist, JTE013 was given orally to mice immediately after reperfusion. JTE013 administration reduced the number of activated microglia and reversed their morphology from amoeboid to ramified microglia in post-ischemic brain after tMCAO challenge, along with attenuated microglial proliferation. Moreover, JTE013 administration attenuated M1 polarization in post-ischemic brain. This S1P_2_-directed M1 polarization appeared to occur in activated microglia, which was evidenced upon JTE013 exposure *in vivo* as suppressed M1-relevant NF-κB activation in activated microglia of post-ischemic brain. Moreover, JTE013 exposure or S1P_2_ knockdown reduced expression levels of M1 markers *in vitro* in lipopolysaccharide-driven M1 microglia. Additionally, suppressing S1P_2_ activity attenuated activation of M1-relevant ERK1/2 and JNK in post-ischemic brain or lipopolysaccharide-driven M1 microglia. Overall, our study demonstrated that S1P_2_ regulated microglial activation and M1 polarization in post-ischemic brain.

## Introduction

Microglia primarily regulate immune responses in the central nervous system (CNS)^1^. They react to brain injury and become activated to play either beneficial or detrimental roles in injured brain^2^. In the latter case, activated microglia are shaped as amoeboid cells^3^ and their phenotypes are rapidly changed into M1-polarized cells^4^, contributing to detrimental immune responses by producing various pro-inflammatory cytokines^5^. These pathogenic features are well characterized in injured brain of cerebral ischemia caused by an insufficient blood flow into the brain^6,7^. For example, microglial activation occurs in both periischemic and ischemic core regions of the brain after a transient focal cerebral ischemia and persists up to many days^8^. Activated microglia at the chronic phase (over 3 days after ischemic challenge) can transform their morphology from ramified to amoeboid in the ischemic core region^8–10^ and proliferate in the marginal zone between periischemic and ischemic core regions^11^. In addition, activated microglia can polarize to pro-inflammatory M1 and anti-inflammatory M2 phenotypes in post-ischemic brain, contributing to brain injury and ischemic recovery, respectively^12^. Given that microglial activation is a core pathogenesis in post-ischemic brain, targeting microglial activation is an emerging strategy to develop therapeutics for treating cerebral ischemia. Many efforts have been made to identify regulators of microglial activation in the post-ischemic brain^6^.

Sphingosine 1-phosphate (S1P), an important bioactive lysophospholipid, regulates a variety of biological functions through its five specific G protein-coupled receptors (S1P_1–5_) that are ubiquitously expressed throughout the body^13,14^. In particular, receptor-mediated S1P signaling has become an emerging drug target to treat cerebral ischemia because of FTY720’ efficacy in human patients^15–17^ and animal models^18–23^. Up to date, two of FTY720 efficacy-relevant S1P receptors, S1P_1_^24^ and S1P_3_^11^, and one FTY720 efficacy-irrelevant S1P receptor, S1P_2_^25^, have been identified to play a role in the pathogenesis of cerebral ischemia. Of note, the pathogenesis of cerebral ischemia *via* S1P_1_ and S1P_3_ is closely linked to microglial activation involving morphological changes into amoeboid cells, proliferation, and production of pro-inflammatory cytokines, a feature of M1 polarization^11,24^. However, it remains unknown whether S1P_2_-directed pathogenesis is linked into microglial activation in post-ischemic brain. It is known that the pathogenic role of S1P_2_ in post-ischemic brain is linked to vascular dysfunction by enhancing MMP-9 activity^25^. Although S1P_2_ is not elucidated as a regulator of microglial activation in post-ischemic brain yet, it can be the regulator since its role in inflammation has been reported for peripheral tissues^26–28^. S1P_2_ on endothelial cells can trigger vascular dysfunction through NF-κB activation that subsequently results in increased production of proinflammatory mediators^28^. S1P_2_ also influences inflammatory atherosclerosis by modulating the production of proinflammatory cytokines (IL-1β and IL-18) and macrophages activation^29^. Additionally, suppressing S1P_2_ activity can attenuate acute renal ischemic injury by downregulating inflammatory cytokines^27^. Therefore, it is possible that S1P_2_ could also regulate neuroinflammatory responses in the brain after ischemic challenge by activating microglia, leading to brain ischemic injury^7,30,31^. In addition, microglia are the main loci for S1P_2_ expression in the brain^32^, suggesting that S1P_2_ could regulate microglial activation in post-ischemic brain. This notion could be supported by a study using another disease model, in which JTE013 attenuated microglial activation and subsequent proinflammatory responses in the brain of mouse with hepatic encephalopathy^33^.

In this study, we aimed to address the relationship between S1P_2_ and microglial activation in view of pathogenesis of cerebral ischemia using transient middle cerebral artery occlusion (tMCAO) in mice. Microglial activation and their morphological changes in post-ischemic brain were analyzed through Iba1 immunohistochemical analysis at both acute (1 day after tMCAO) and chronic phases (3 days after tMCAO). Moreover, we analyzed microglial proliferation and phenotypic transition, likely M1/M2 polarization, in post-ischemic brain. To demonstrate microglia as a responsible cell type for the latter, we examined *in vivo* cell polarization-relevant microglial NF-κB activation in post-ischemic brain and *in vitro* expression levels of cell polarization markers in BV2 microglia cell line using an inducer of M1 polarization, lipopolysaccharide (LPS). Finally, we determined M1- and S1P_2_-relevant downstream effector signaling in post-ischemic brain *in vivo* as well as LPS-activated BV2 microglia *in vitro*.

## Results

### Suppressing S1P_2_ activity attenuates microglial activation and proliferation in post-ischemic brain after tMCAO challenge

**Figure 1:**
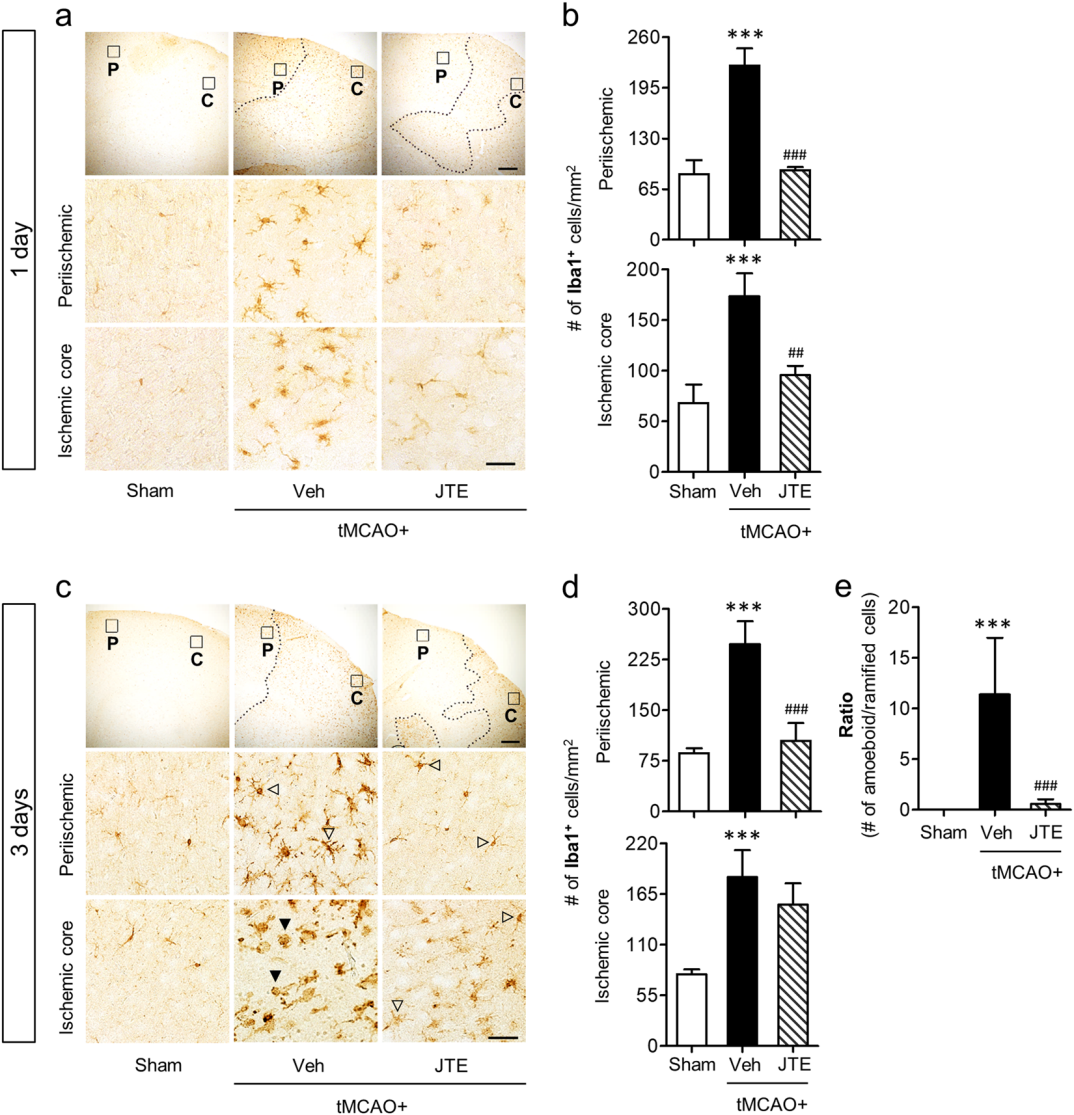
Suppressing S1P_2_ activity reduces the number of Iba1-immunopositive cells in post-ischemic brain at 1 day and 3 days after tMCAO challenge. Effects of JTE013 (JTE) on microglia activation were observed by Iba1 immunohistochemical staining at 1 day (**a**,**b**) and 3 days (**c**–**e**). (**a**,**c**) Representative photographs of Iba1-immunopositve cells in periischemic (P) and ischemic core (C) regions at 1 day (**a**) and 3 days (**c**). Diagram box in the upper panel shows the cerebral area where images in middle and bottom panels are obtained. Dotted lines separate periischemic and ischemic core regions. Open arrowheads indicate ramified microglia and closed arrowheads indicate amoeboid microglia (**c**). Scale bars, 200 µm (top) and 50 µm (middle and bottom). (**b**,**d**,**e**) Quantification of Iba1 immunopositive cells in cells per mm^2^ in both regions (**b**,**d**) and the ratio of amoeboid to ramified microglia (**e**). n = 4–5 mice per group. ^***^p < 0.001 vs. sham; ^##^*p* < 0.01 and ^###^*p* < 0.001 vs. vehicle-treated group by Newman-Keuls multiple range test.

Microglia activation is a core pathogenic feature in injured brain by ischemic challenge. Microglia become activated in both the ischemic core and periischemic regions and their morphology in the ischemic core region is converted into highly detrimental type, amoeboid microglia, especially in the chronic phase (likely at least 3 days after the ischemic challenge)^8–10^. S1P_2_ is a pathogenic factor for brain damage in cerebral ischemia^25^. Thus, we investigated whether microglial activation might play a role in S1P_2_-mediated brain damage in cerebral ischemia. We first determined microglial activation in both periischemic and the ischemic core regions of post-ischemic brain at 1 and 3 days after tMCAO challenge through immunohistochemical analysis for Iba1, a well-known marker for activated microglia^8,34^. The tMCAO challenge caused a marked increase in the number of Iba1-immunopositive cells in both periischemic and ischemic core regions at 1 day after tMCAO (Fig. 1a,b). This robust increase was significantly attenuated when S1P_2_ activity was suppressed by oral administration of JTE013, a specific S1P_2_ antagonist, immediately after reperfusion (Fig. 1a,b). In the chronic phase (3 days after tMCAO), the number of Iba1-immunopositive cells was increased in both regions of the injured brain (Fig. 1c,d). Suppressing S1P_2_ activity by JTE013 administration resulted in a region-specific reduction in the number of Iba1-immunopositive cells (Fig. 1c,d). JTE013 administration significantly reduced the number of Iba1-positive cells in the periischemic region, but not in the ischemic core region at 3 days after tMCAO (Fig. 1c,d). Interestingly, in the ischemic core region at this time point, S1P_2_ was associated with morphological changes of Iba1-immunopositive cells from ramified to amoeboid cells, another feature of microglial activation^8,9^. In fact, most Iba1-immunopositive cells were shaped as amoeboid in the ischemic core region at 3 days after tMCAO (Fig. 1c,e). However, after JTE013 administration, most of Iba1-immunopositive cells were shaped as ramified in the same region (Fig. 1c,e). These results clearly demonstrate that S1P_2_ is a critical regulator for microglial activation in cerebral ischemia. In addition, we can affirm that pharmacological suppression of S1P_2_ activity reduced brain damage at 1 and 3 days after tMCAO as evidenced by reduced neural cell death (Supplementary Fig. 1a,c) and improved neurological functions (Supplementary Fig. 1b,d) through FJB staining and mNSS scoring. Taken together, these results indicate that S1P_2_-regulated microglial activation could be associated with ischemic brain injury.

**Figure 2:**
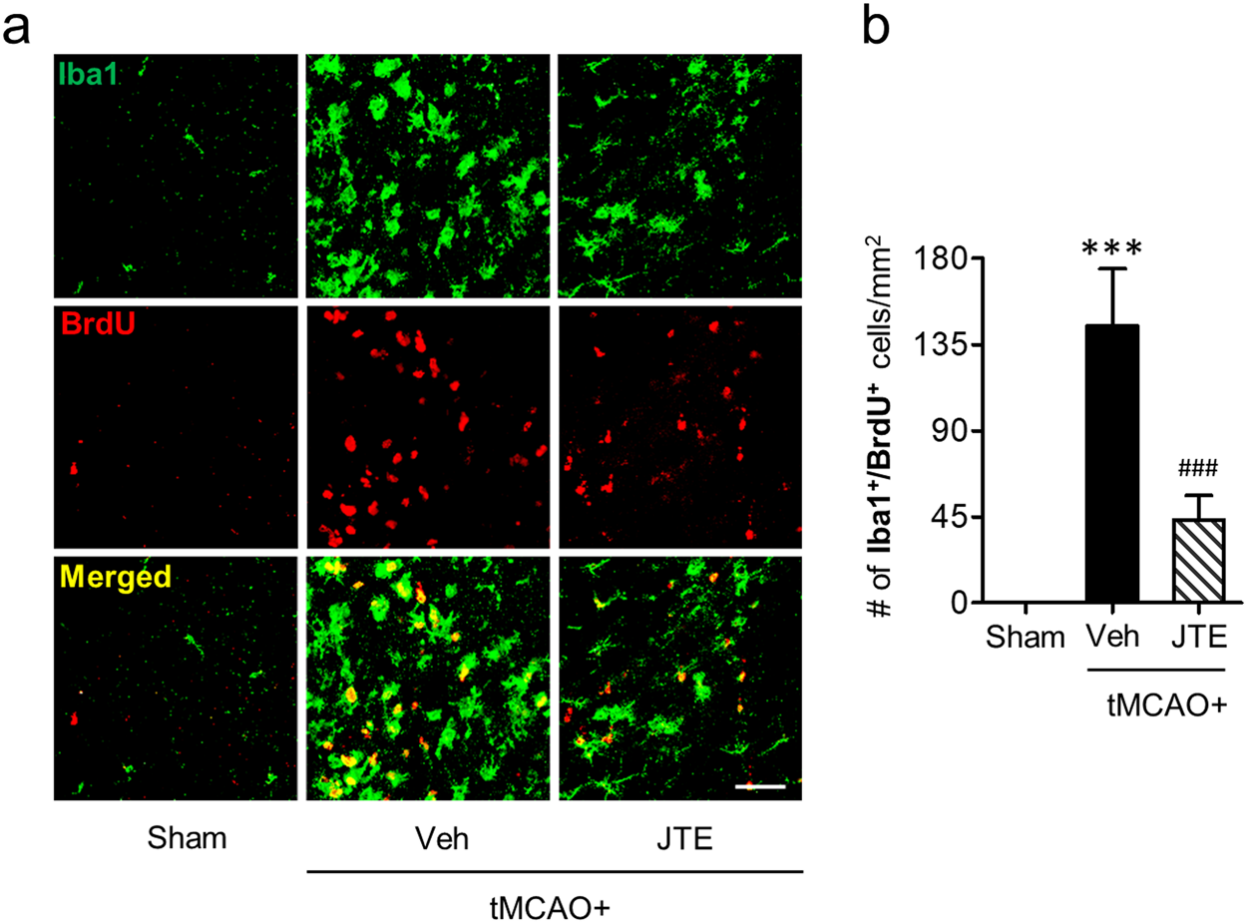
Suppressing S1P_2_ activity attenuates microglial proliferation in post-ischemic brain. Brain samples from sham, tMCAO, and tMCAO mice exposed to JTE013 (JTE) were used to determine the proliferation of microglial cell through Iba1 and BrdU double immunohistochemical labelling at 3 days after tMCAO challenge. (**a**) Representative photographs of Iba1 and BrdU immunopositive cells in penumbra regions (region between periischemic and ischemic areas). Scale bar, 50 µm. (**b**) Quantification of the number of Iba1 and BrdU double immunopositive cells in cells per mm^2^. n = 5 mice per group. ***p < 0.001 vs. sham; ^###^*p* < 0.001 vs. vehicle-treated group by Newman-Keuls multiple range test.

Activated microglia can proliferate in the injured brain after ischemic challenge. They are generally observed in the marginal zone between periischemic and ischemic core regions. Thus, we investigated whether S1P_2_ also affected microglial proliferation in the marginal zone of the brain after tMCAO through *in vivo* BrdU incorporation and subsequent analysis of Iba1/BrdU double immunofluorescence staining. In vehicle-treated tMCAO mice, the number of Iba1/BrdU-double immunopositive cells was greatly increased in the marginal zone of post-ischemic brain (Fig. 2). However, JTE013 administration immediately after tMCAO challenge significantly attenuated such increase by approximately 70% (Fig. 2). These data demonstrate that S1P_2_ is also involved in microglial proliferation in post-ischemic brain.

### Suppressing S1P_2_ activity attenuates microglial M1 polarization in post-ischemic brain after tMCAO challenge

**Figure 3:**
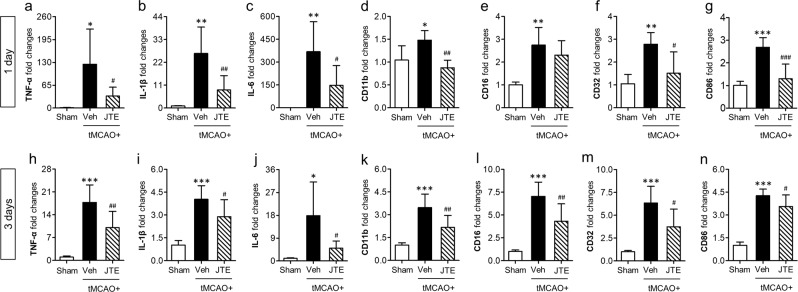
Suppressing S1P_2_ activity downregulates mRNA expression levels of markers for M1 polarization in post-ischemic brain. Ipsilateral brain hemispheres from sham, tMCAO, and tMCAO mice exposed to JTE013 (JTE) were used to analyze changes in expression level of M1 markers including TNF-α, IL-1β, IL-6, CD11b, CD16, CD32, and CD86 by qRT-PCR analysis at 1 day (**a**–**g**) and 3 days (**h**–**n**) after tMCAO challenge. n = 5–6 mice per group. *p < 0.05, ^**^p < 0.01, and ^***^p < 0.001 vs. sham; ^#^*p* < 0.05, ^##^*p* < 0.01, and ^###^*p* < 0.001 vs. vehicle-treated group by Newman-Keuls multiple range test.

**Figure 4:**
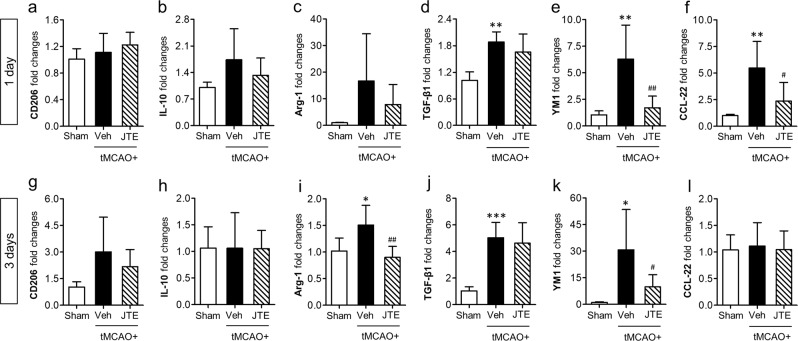
Suppressing S1P_2_ activity does not enhance mRNA expression levels of markers for M2 polarization in post-ischemic brain. Ipsilateral brain hemispheres from sham, tMCAO, and tMCAO mice exposed to JTE013 (JTE) were used to analyze changes in expression level of M2 markers, including CD206, IL-10, Arg-1, TGF-β1, YM1, and CCL-22 by using qRT-PCR analysis at 1 day (**a**–**f**) and 3 days (**g**–**l**) after tMCAO challenge. n = 5–6 per group. ^*^p < 0.05, ^**^p < 0.01, and ^***^p < 0.001 vs. sham; ^#^*p* < 0.05 and ^##^*p* < 0.01 vs. vehicle-treated group by Newman-Keuls multiple range test.

A phenotypical change in microglial polarization with M1/M2 is an important aspect of immune responses in diverse CNS diseases^35,36^. In cerebral ischemia, microglia also become rapidly polarized into pro-inflammatory M1 and anti-inflammatory M2 phenotypes^30^. To determine whether S1P_2_ signaling could direct microglial M1/M2 polarization, we performed qRT-PCR analysis primarily for brain samples obtained at 1 and 3 days after tMCAO. All determined soluble markers (TNF-α, IL-1β, and IL-6) and surface markers (CD11b, CD16, CD31, and CD86) for M1 polarization were upregulated in the brain challenged by tMCAO at both 1 and 3 days (Fig. 3). Such enhanced M1 polarization in post-ischemic brain was significantly attenuated by suppressing S1P_2_ activity with JTE013 administration (Fig. 3). These data clearly demonstrate that S1P_2_ signaling directs pro-inflammatory M1 polarization in post-ischemic brain. However, suppressing S1P_2_ activity did not enhance anti-inflammatory M2 phenotypes, which was determined by measuring mRNA expression levels of M2 markers (CD206, IL-10, Arg-1, TGF-β1, YM1, and CCL-22) (Fig. 4). In some cases, it rather reduced mRNA expression levels of M2 markers (YM1 and CCL-22 in 1-day post-ischemic brain and Arg-1 and YM1 in 3-day post-ischemic brain). Altogether, these results demonstrate that S1P_2_ signaling skews microglia towards mostly M1 polarization in injured brain after ischemic challenge.

**Figure 5:**
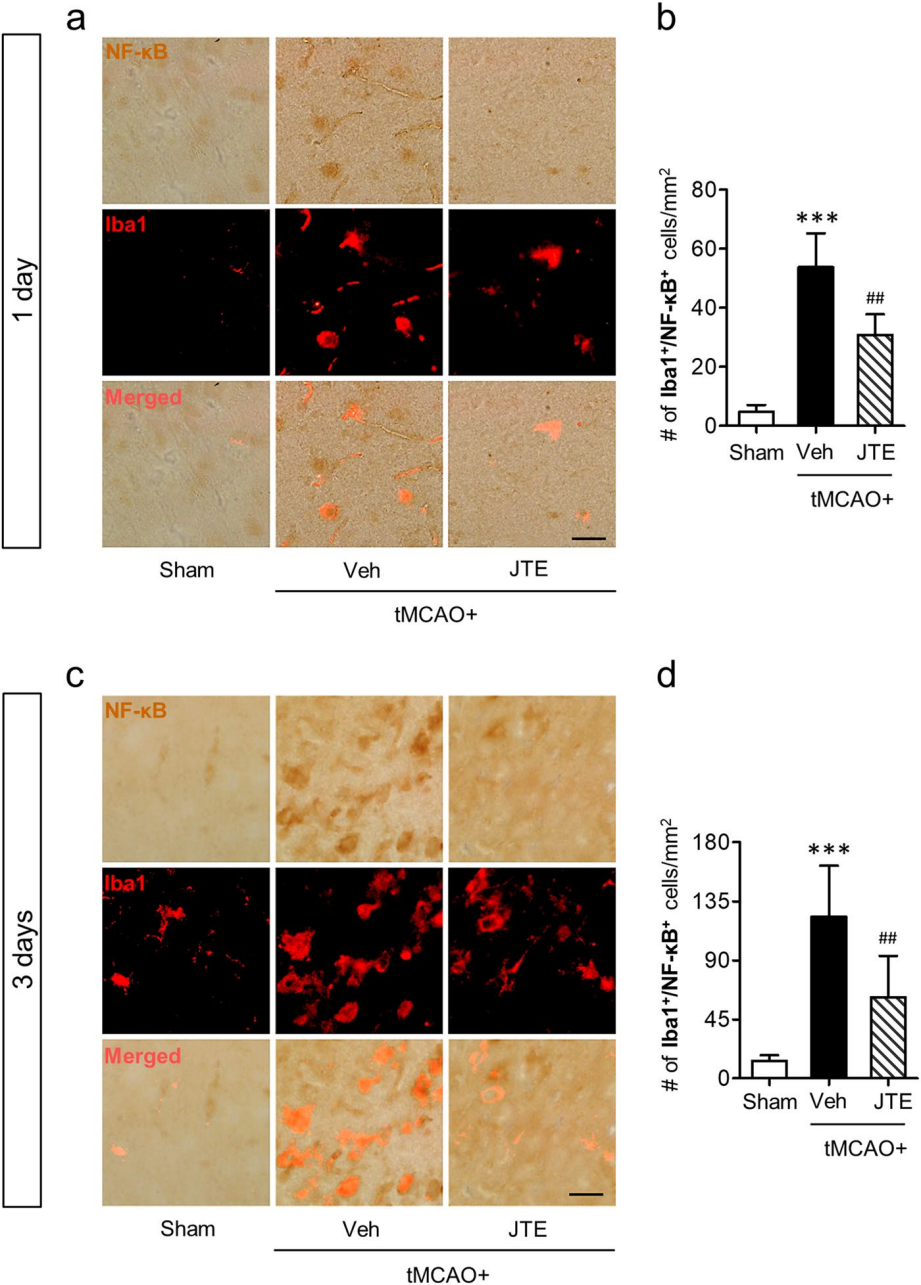
Suppressing S1P_2_ activity attenuates microglial NF-κB activation in post-ischemic brain. Effects of JTE013 (JTE) on NF-κB expression in activation microglia were observed by Iba1/NF-κB double immunohistochemistry. (**a**,**c**) Representative photographs of Iba1/NF-κB double immunopositive cells in the post-ischemic brain at 1 day (**a**) and 3 days (**c**) after tMCAO challenge. (**b**,**d**) Quantification of the number of Iba1/NF-κB double immunopositive cells in cells per mm^2^ at 1 day (**b**) and 3 days (**d**) after tMCAO challenge. n = 4–5 mice per group. Scale bar, 50 µm. ^***^p < 0.001 vs. sham; ^##^*p* < 0.01 vs. vehicle-treated group by Newman-Keuls multiple range test.

In cerebral ischemia, M1 polarization mainly occurs in activated microglia^11,30^. Therefore, we next investigated whether the currently revealed S1P_2_-driven M1 polarization in post-ischemic brain occurred in activated microglia by determining a critical factor for M1 polarization, NF-κB activation, in activated microglia^11,31^. For this, we employed NF-κB(p65)/Iba1 double-immunofluorescence staining. As expected, NF-κB was activated in the brain both at 1 day (Fig. 5a,b) and 3 days (Fig. 5c,d) after tMCAO challenge. Most NF-κB(p65) signals were observed in Iba1-immunepositive cells (Fig. 5), strongly demonstrating that activated microglia were the loci for M1 polarization in post-ischemic brain. This NF-κB activation in activated microglia was significantly attenuated by JTE013 administration (Fig. 5), further demonstrating that S1P_2_ could regulate M1 microglial polarization in post-ischemic brain.

**Figure 6:**
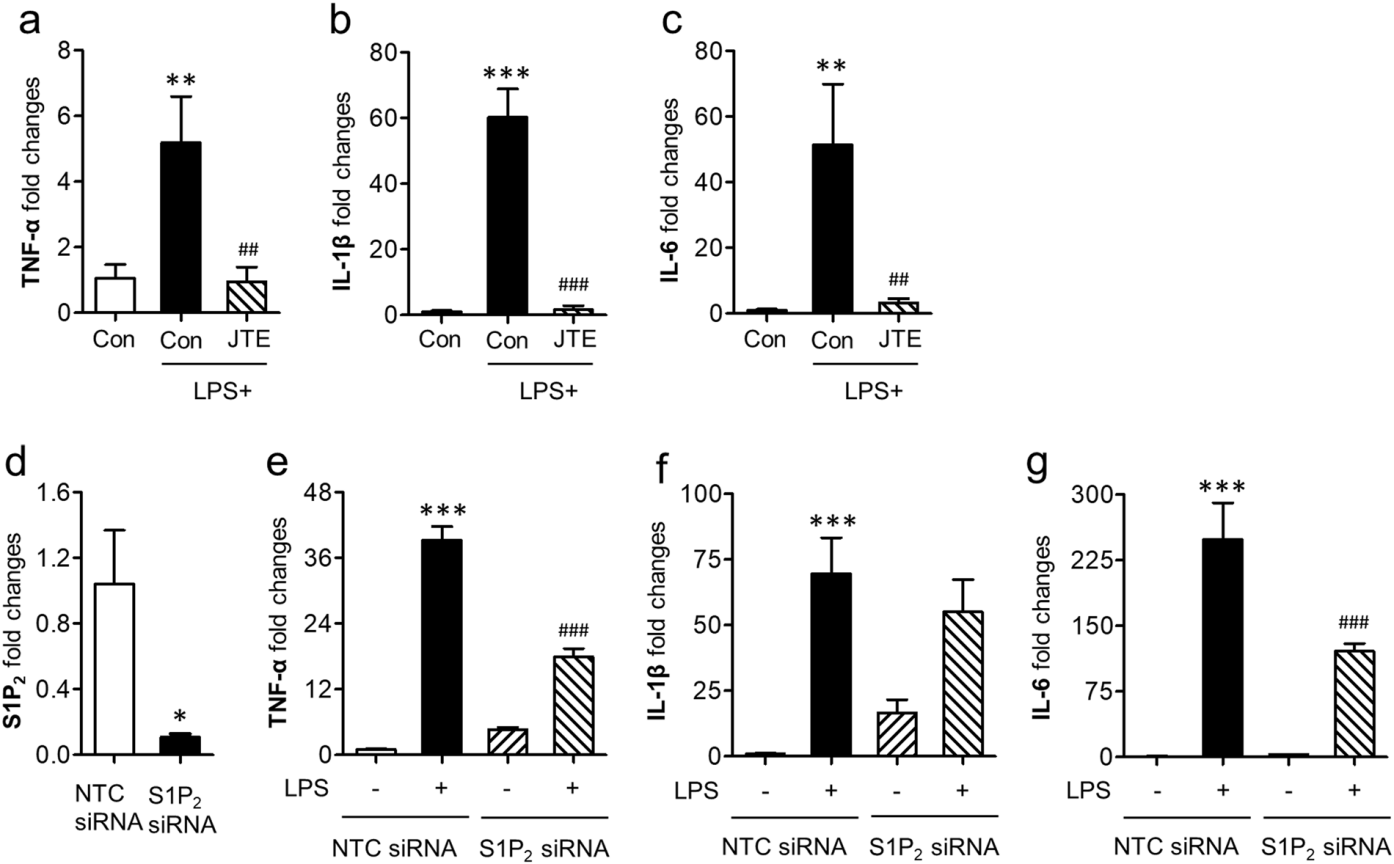
Suppressing S1P_2_ activity downregulates mRNA expression levels of soluble markers for M1 polarization in LPS-stimulated BV2 microglia. Effects of JTE013 (JTE) (**a**–**c**) on LPS (100 ng/ml)-stimulated BV2 cells were determined by analyzing changes in expression level of M1 markers including TNF-α (**a**), IL-1β (**b**), and IL-6 (**c**) by qRT-PCR analysis. n = 3 per group. **p < 0.01 and ^***^p < 0.001 vs. control (con); ^##^*p* < 0.01 and ^###^*p* < 0.001 vs. LPS-treated group by Newman-Keuls multiple range test. (**d**) Knockdown efficiency for S1P_2_ was determined by qRT-PCR. n = 3 per group. ^*^p < 0.05 vs. non-target control siRNA (NTC siRNA) by Mann-Whitney test. Effects of S1P_2_ knockdown (**e**–**g**) on LPS (100 ng/ml)-stimulated BV2 cells were determined by analyzing changes in expression level of M1 markers including TNF-α (**e**), IL-1β (**f**), and IL-6 (**g**) by qRT-PCR analysis. n = 3 per group. ^***^p < 0.001 vs. NTC siRNA; ^###^*p* < 0.001 vs. LPS-treated grou*p* infected with NTC siRNA (NTC siRNA + LPS) by Newman-Keuls multiple range test.

The current *in vivo* study demonstrated that S1P_2_ could direct M1 polarization in cerebral ischemia. To reaffirm this notion *in vitro*, we used BV2 microglia cell line and treated cells with LPS, a well-known inducer of M1 polarization^5,37^. Upon LPS stimulation, mRNA levels of M1 soluble markers such as TNF-α, IL-1β, and IL-6 were upregulated in BV2 microglia (Fig. 6a–c). However, they were markedly attenuated by JTE013 exposure (Fig. 6a–c). Similarly, S1P_2_ knockdown (Fig. 6d) attenuated mRNA expression levels of M1 soluble markers, except IL-1β (Fig. 6e–g). These *in vitro* data clearly demonstrate that S1P_2_ is a critical factor for skewing microglia into M1 phenotypes, further supporting that S1P_2_ could contribute to ischemic brain injury by directing microglial M1 polarization.

### Suppressing S1P_2_ activity attenuates M1-relevant ERK1/2 and JNK MAPKs phosphorylation in post-ischemic brain and LPS-stimulated BV2 microglia

**Figure 7:**
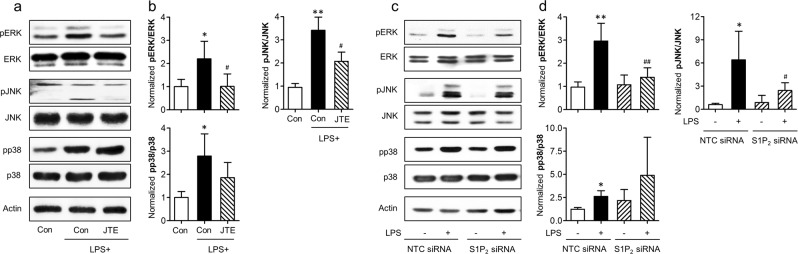
Suppressing S1P_2_ activity attenuates phosphorylation of M1-relavant ERK1/2 and JNK MAPKs in LPS-stimulated BV2 microglia. Effects of JTE013 (JTE) (**a**,**b**) or S1P_2_ knockdown (**c**,**d**) on LPS (1 µg/ml)-stimulated BV2 cells were determined by analyzing phosphorylation of ERK1/2, JNK, and p38 MAPKs through Western blot analysis. (**a**,**c**) Representative Western blots of each MAPK. (**b**,**d**) Quantification (n = 3 per group). ^*^p < 0.05 and ^**^p < 0.01 vs. control group (con, b) or control group transfected with non-target control siRNA (NTC siRNA, **d**); ^#^*p* < 0.05 and ^##^*p* < 0.01 vs. LPS-treated group (**b**) or LPS-treated group transfected with NTC siRNA (NTC siRNA + LPS, **d**) by Newman-Keuls multiple range test.

**Figure 8:**
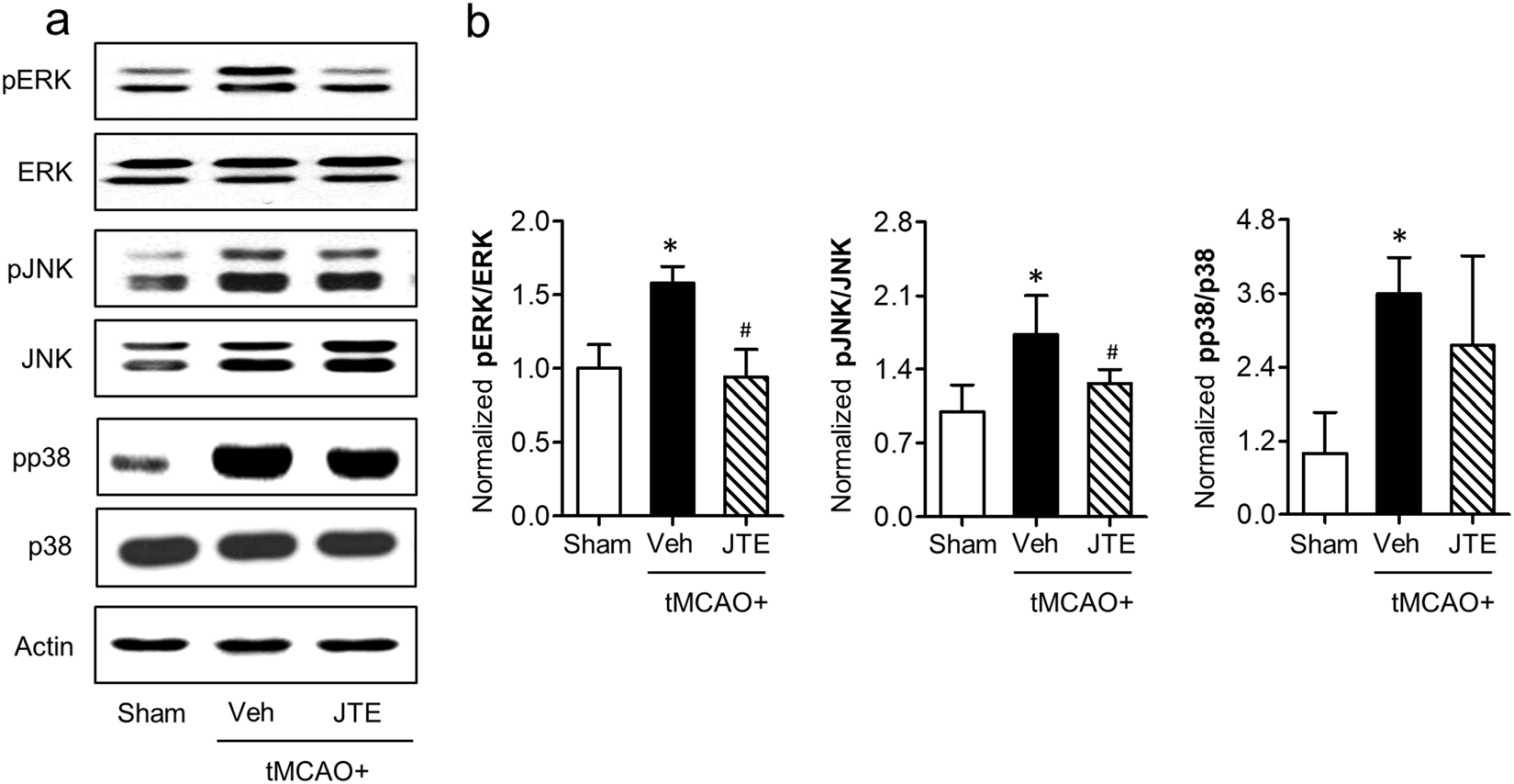
Suppressing S1P_2_ activity attenuates phosphorylation of M1-relavant ERK1/2 and JNK MAPK in post-ischemic brain. Ipsilateral brain hemispheres from sham, tMCAO, and tMCAO mice exposed to JTE013 (JTE) were used to analyze phosphorylation of ERK1/2, JNK, and p38 MAPKs by Western blot analysis. (**a**) Representative Western blots of each MAPK. (**b**) Quantification (n = 4 mice per group). ^*^p < 0.05 vs. sham; ^#^*p* < 0.05 vs. vehicle-treated group by Newman-Keuls multiple range test.

The current *in vivo* and *in vitro* findings demonstrated that S1P_2_ could drive M1 polarization in post-ischemic brain. Among S1P_2_-triggered effector pathways, G_i_-mediated MAPKs signaling pathways are well known effector pathways for M1 polarization^38–40^. Therefore, we sought to determine whether MAPKs might be involved in S1P_2_-driven M1 polarization. When BV2 microglia were polarized into M1 phenotype with LPS exposure, all three MAPKs (ERK1/2, p38 MAPK, and JNK MAPK) were activated as evidenced by their increased phosphorylation (Fig. 7a,b). Interestingly, ERK1/2 and JNK MAPK, but not p38 MAPK, were influenced by S1P_2_. Suppressing S1P_2_ activity by JTE013 exposure significantly attenuated the phosphorylation of ERK1/2 and JNK (Fig. 7a,b), which was reaffirmed upon S1P_2_ knockdown (Fig. 7c,d). These *in vitro* data demonstrated that S1P_2_ enhanced phenotypical changes of microglia into M1 polarization through activating its effector pathways, ERK1/2 and JNK. We further determined whether these effector pathways were regulated by S1P_2_ in post-ischemic brain. In the vehicle-treated group, phosphorylation levels of all three MAPKs (ERK1/2, p38, and JNK) were significantly increased in post-ischemic brain (Fig. 8a,b). Suppressing S1P_2_ activity by JTE013 administration significantly attenuated the phosphorylation of ERK1/2 and JNK, but not that of p38 (Fig. 8a,b). These *in vivo* data demonstrated that S1P_2_ influenced M1 polarization through ERK1/2 and JNK pathways in post-ischemic brain.

## Discussion

S1P receptors have been revealed as valuable therapeutic targets to treat cerebral ischemia due to successful results in validating FTY720’s efficacy in human patients and animal models. Along with this success, some S1P receptor subtypes that can mediate pathogenesis of cerebral ischemia have been identified, including S1P_1_^24^ and S1P_3_^11^, both of which are target receptor subtypes of FTY720^41^. In addition to FTY720-relevant receptor subtypes, S1P_2_ that is not a target receptor subtype of FTY720 has been revealed as a pathogenic factor for cerebral ischemia^25^. Thus, up to now, three different S1P receptor subtypes have been identified to contribute to brain injury in cerebral ischemia. In view of pathogenic mechanisms, it has been reported that these receptors can mediate different pathogenesis. S1P_1_ and S1P_3_ regulate microglial activation to act their detrimental roles in an ischemic brain^11,24^ while S1P_2_ mediates cerebrovascular dysfunction^25^. However, the identified pathogenesis might be part of diverse mechanisms. In fact, S1P_1_ is also involved in cerebrovascular dysfunction after ischemic challenge^24^. S1P_3_ is also associated with impaired vascular permeability in brain tumor^42^. Therefore, there might be more diverse mechanisms associated with each S1P receptor-dependent brain injury in cerebral ischemia. In this context, S1P_2_ could trigger another pathogenic event in post-ischemic brain in addition to cerebrovascular dysfunction. This was addressed in the present study. The current study revealed that S1P_2_ in post-ischemic brain was closely associated with microglial activation, a core pathogenesis in cerebral ischemia. Suppressing S1P_2_ activity by JTE013 administration attenuated microglial activation, proliferation, morphological changes into toxic amoeboid shape, and M1 polarization, all of which indicated that S1P_2_ could drive microglia toward detrimental functions, leading to brain damage.

Receptor-mediated S1P_2_ signaling is well characterized in inflammatory diseases, both in peripheral tissues and CNS^25–28,43^. S1P_2_ aggravates disease pathogenesis of bleomycin-induced pulmonary fibrosis by producing proinflammatory mediators, including TNF-α, IL-1β, IL-6, and monocyte chemoattractant protein (MCP)^26^. Similar proinflammatory roles of S1P_2_ have been reported in renal ischemic injury, in which S1P_2_ antagonism with JTE013 administration can protect renal cells by downregulating mRNA expression of proinflammatory mediators such as TNF-α, ICAM-1, MCP-1, and MIP-2^27^. S1P_2_ is also associated with vascular inflammation as it increases vascular permeability through endothelial NF-κB activation^28^. These independent studies emphasizing proinflammatory roles of S1P_2_ in peripheral tissues indicate that S1P_2_ could modulate inflammatory cascades in post-ischemic brain as well. Indeed, recent reports have suggested that S1P_2_ plays critical roles in aggravating neuroinflammation of post-ischemic brain through neurovascular inflammation^25,43^. Despite this clear link between S1P_2_ and ischemia-induced neuroinflammation, whether S1P_2_ regulates microglial activation, a key inflammatory event in post-ischemic brain, has not been revealed yet. The current study addressed this issue and provided a novel S1P_2_-mediated event, S1P_2_-regulated microglial activation in post-ischemic brain. Suppressing S1P_2_ activity dramatically reduced the number of activated microglia, decreased proliferation of activated microglia, and reversed morphology of activated microglia from amoeboid cells into ramified cells in post-ischemic brain after tMCAO challenge. Therefore, based on previous and current findings, we can conclude that S1P_2_ contributes to brain injury after ischemic challenge by aggravating immune responses in the brain through both vascular inflammation^25,43^ and microglial activation (the current study). The latter was further supported by a recent study, demonstrating that suppressing S1P_2_ activity by JTE013 exposure attenuated microglial activation and reduced proinflammatory responses in the cortex of mouse with hepatic encephalopathy^33^. Interestingly, these inflammatory roles have also been reported for S1P_1_ and S1P_3_. S1P_1_ and S1P_3_ can trigger ischemic pathogenesis through microglial activation^11,24^ and vascular inflammation^24,42^ in post-ischemic brain, similar to S1P_2_ (vascular inflammation^25,43^ and microglial activation (current study)). In this notion, these three identified S1P receptor subtypes (S1P_1_, S1P_2_, and S1P_3_) as pathogenic factors for cerebral ischemia might share common pathogenesis for ischemic injury in the brain, likely neuroinflammation.

A critical feature of neuroinflammatory diseases including cerebral ischemia is that M1 and M2 polarization of immune cells can exert distinct responses: neurotoxic and neuroprotective. Particularly, increased number of amoeboid microglia is a strong indication for phenotypical changes into M1 polarization in injured brain because amoeboid microglia can act as detrimental cells to neurons following ischemic injury by secreting M1-relevant inflammatory mediators^44,45^. The current study revealed that S1P_2_ was involved in triggering morphological transformation of microglia toward amoeboid shape, indicating that S1P_2_ could mediate M1 microglial polarization. Indeed, we further demonstrated that S1P_2_ triggered M1 polarization in post-ischemic brain as evidenced by attenuated mRNA upregulation of M1 surface and soluble markers upon suppressing S1P_2_ activity. Importantly, activated microglia seem to be the main loci for this S1P_2_-regulated M1 polarization. NF-κB activation, a key mechanism for M1 polarization^31,46^, was observed mostly in activated microglia of post-ischemic brain. It was attenuated by suppressing S1P_2_ activity in this study. This *in vivo* role of S1P_2_ in M1 microglial polarization was further confirmed in BV2 murine microglia stimulated by using LPS, an inducer of M1 polarization. In these cells, suppressing S1P_2_ activity significantly downregulated expression of functionally important M1 markers (TNF-α, IL-1β, and IL-6). Of note, S1P_2_ seems to influence even more M1 polarization than M2 polarization in post-ischemic brain. In this study, suppressing S1P_2_ activity did not show significant effects on M2 polarization in post-ischemic brain, although a few M2 markers were unexpectedly downregulated when S1P_2_ activity was inhibited, indicating that S1P_2_ could be mainly associated with promoting inflammatory M1 polarization rather than anti-inflammatory M2 polarization following ischemic injury. This notion can be further supported by a recent report suggesting the role of S1P_2_ in macrophage polarization in peripheral tissues, in which suppressing S1P_2_ activity attenuated M1 polarization of macrophages following liver injury^47^. Therefore, pathogenic roles of S1P_2_ in post-ischemic brain can be mainly associated with suppression of inflammatory cascades through influencing M1 polarization rather than ischemic damage repair through M2 polarization.

Receptor-mediated S1P signaling exerts pleotropic biological functions in different organs through intracellular effector pathways^13^. Among them, S1P receptors can commonly activate G_αi_-downstream signaling pathways that are mainly associated with M1/M2 polarization^38–40,48^. In particular, G_αi_-mediated activation of MAPKs is closely associated with M1 polarization through transcriptional activation of NF-κB^38–40^. Similarly, in this study, S1P_2_-regulated M1 polarization in activated microglia seemed to be mediated by activation of ERK1/2 and JNK MAPKs. Suppressing S1P_2_ activity attenuated phosphorylation of ERK1/2 and JNK, but not that of p38 MAPK, in activated BV2 microglia towards M1 cells by LPS stimulation. Furthermore, in post-ischemic brain, activation of these two signaling molecules were dependent on S1P_2_. This might be the underlying mechanism for S1P_2_ to regulate M1 polarization in post-ischemic brain.

Roles of S1P_2_ in the current study was identified by the use of JTE013 because it has been known as an antagonist for S1P_2_^49,50^. However, JTE013 could act on additional types of target. Its effects include agonist at S1P_1_^51^, antagonist at S1P_4_^52^, and blockers for other non-receptor targets^53^. Considering these previous reports, the currently observed effects of JTE013 could be mediated through its action at other targets. However, JTE013’s effects might be irrelevant to S1P_1_ activation because S1P_1_ knockdown in the brain attenuated brain damage and microglial activation^24^. If JTE013 acted as an agonist for S1P_1_, it should potentiate brain damage and microglial activation. However, JTE013 administration reduced microglial activation (the current study) and brain damage following ischemic challenge (Kim *et al*.^25^; the current study). It might be also considerable that synthetic S1P_1_ agonists such as FTY720 and AUY954 act as functional antagonists to S1P_1_^54,55^ and exert anti-inflammatory effects^56,57^. The observed effects of JTE013 in the current study might be mediated, in part, through S1P_1_ if JTE013 acted as a functional antagonist to S1P_1_. Roles of S1P_4_ in either cerebral ischemia or microglial activation remain unidentified, and therefore, it is unclear that JTE013 could exert its effects by acting on S1P_4_. JTE013 might also exert its effects by inhibiting KCl or endothelial thromboxane-1^53^ because these two molecules were known to regulate ischemic brain damage^58,59^. Although we could not exclude possible involvement of other targets, it is clear that, at least, S1P_2_ is involved in microglial activation and M1 polarization. In the current *in vitro* study, either JTE013 exposure or S1P_2_ knockdown attenuated LPS-induced microglial M1 polarization and MAPKs phosphorylation. In addition, the current and previous *in vivo* studies have shown that either JTE013 treatment (the current study; Kim *et al*.^25^) or S1P_2_ deletion in mice^25^ reduces brain damage following ischemic challenge. These two independent studies support that JTE013’s effects may be mediated through S1P_2_ antagonism, further suggesting S1P_2_ as a main player for microglial activation and M1 polarization after tMCAO challenge.

Iba1 has been widely used as a microglial marker for immunohistochemical analysis, but it can also be used as a marker for non-microglial cells, including monocyte/macrophages. In particular, robust infiltration of macrophages occurs in the ischemic core region at 3 days after tMCAO^60,61^ because of blood brain barrier (BBB) disruption. Therefore, in the current study, Iba1-immunopositive cells in the ischemic core region at 3 days after tMCAO could be either activated microglia or infiltrated macrophages. However, many of the currently observed Iba1-immunopositive cells upon JTE013 administration could be more likely activated microglia. In fact, JTE013 administration after tMCAO remarkably attenuated BBB disruption^25^, suggesting that JTE013 administration could reduce macrophage infiltration into the ischemic core region. In contrast, JTE013 administration did not reduce total number of Iba1-immunopositive cells in the ischemic core region at 3 days after tMCAO, suggesting that most of the Iba1-immunopositive cells would be activated microglia rather than infiltrated macrophages.

In conclusion, we provided a new role of S1P_2_ in ischemic pathogenesis such as microglial activation and M1 polarization, along with activation of its effector pathways including ERK1/2 and JNK. This study may provide a complementary pathogenic role of S1P_2_ in cerebral ischemia in addition to previously provided roles of S1P_2_ in neurovascular inflammation^25,43^. Up to now, three of five S1P receptor subtypes (S1P_1_^24^, S1P_2_ (the current study), and S1P_3_^11^) have been identified as pathogenic factors for cerebral ischemia, all of which can influence microglial activation in post-ischemic brain. In particular, S1P_2_ and S1P_3_ could influence primarily M1 polarization in post-ischemic brain and activated microglia, whereas S1P_1_ influenced both M1 and M2 polarization^57^. These S1P receptor subtypes can activate effector pathways in post-ischemic brain with a slightly different way. S1P_1_ activates all 3 MAPKs (ERK1/2, p38, and JNK). S1P_2_ activates ERK1/2 and JNK while S1P_3_ activates ERK1/2 and p38 MAPK. Besides these three S1P receptor subtypes, it could not be excluded for remaining S1P receptor subtypes to be involved in ischemia-induced microglial activation and brain damage in cerebral ischemia.

## Methods

### Animals

All animal experiments and handling were carried out under Center of Animal Care and Use (CACU) guidelines of Lee Gil Ya Cancer and Diabetes Institute (LCDI) at Gachon University, Korea (approved animal protocol number: LCDI-2017-0002). Male ICR mice (6 weeks old) were purchased from Orient Bio (GyeongGi-do, Korea).

### Transient focal cerebral ischemia challenge and drug administration

Male ICR mice (7 weeks old) were used for transient middle cerebral artery occlusion (tMCAO) as previously described^24^. Briefly, mice were anesthetized with 3% isoflurane in 70% N_2_O:30% O_2_ for induction and 1.5% for maintenance. Right middle cerebral artery (MCA) was occluded for 90 min by inserting a 9-mm-long 5-0 monofilament from the bifurcation of the common carotid artery to the MCA. Blood flow was restored by withdrawing the monofilament after 90 min. For the sham-operated group, the same surgical process was applied except for the occlusion. Mice that were subjected to tMCAO were randomly assigned into vehicle-treated and JTE013-treated groups. JTE013 (Cayman Chemical, MI, USA) was dissolved in 2% 2-hydroxypropyl β-cyclodextrin (Sigma-Aldrich, St. Louis, MO, USA) in saline and given orally to mice at 30 mg/kg immediately after reperfusion. The dose of JTE013 was chosen based on a previous study^25^. For the vehicle-treated group, equal volume of the vehicle was given. Four mice with hemorrhage were excluded for any data analysis throughout this study. In addition, three mice died within 24 h following tMCAO challenge: one mouse was for a group of 1 day after tMCAO and two mice were for a group of 3 days after tMCAO.

### Neurological score analysis

Functional neurological deficit was determined by modified neurological severity score (mNSS) 1 or 3 days after tMCAO. The mNSS was determined by motor, sensory, balance, and reflex tests with a total score ranging from 0 to 18 as described previously^24,62^.

### Tissue preparation for histological evaluation

At 1 or 3 days after tMCAO, mice were anesthetized with a mixture of Zoletil 50^®^ (Virbac Laboratories, Carros, France) and Rompun^®^ (Bayer HealthCare LLC, Kansas 66201 U.S.A.) and perfused with ice-cold phosphate-buffered saline (PBS) followed by 4% paraformaldehyde (PFA) solution. Brains were removed, additionally fixed with 4% PFA, and cryoprotected with 30% sucrose solution. They were embedded in Tissue-Tek^®^ optimal cutting temperature compound, frozen on dry ice, and cut into 20 μm sections. To ensure anatomical similarities of brain regions in different experimental groups, two coronal brain sections obtained from the rostral to middle area of the striatum were used in histological experiments. For RNA and protein expression study, mice brains were transcardially washed with ice-cold PBS and ipsilateral brain hemispheres were harvested in liquid nitrogen.

### Fluoro-Jade B staining

Neural cell death after tMCAO was determined by Fluoro-Jade B (FJB) staining. Sections were rinsed with water, incubated in alcohol series (100%, 70%, and 30%), and washed with water. They were oxidized with 0.06% potassium permanganate, rinsed with water, and stained with 0.001% FJB solution containing 0.1% acetic acid. These stained sections were washed with water, dried on a slide warmer, dehydrated with xylene, and mounted with Entellan medium.

### Iba1 immunohistochemistry staining

To determine microglial activation at 1 or 3 days after tMCAO, brain sections were washed with PBS, treated with 1% H_2_O_2_, blocked with 1% fetal bovine serum (FBS) in 0.3% Triton X-100, and labeled with rabbit anti-Iba1 antibody (1:500, Wako Pure Chemicals, Osaka, Japan) overnight at 4 °C. Sections were incubated with a biotinylated secondary antibody (1:200, Santa Cruz, USA) and further incubated with avidin/biotin complex (ABC, 1:100, Vector Laboratories, Burlingame, CA, USA). To develop Iba1 signals, sections were treated with 0.02% 3,3′-diaminobenzidine tetrahydrochloride hydrate (DAB) (Sigma-Aldrich, St. Louis, MO, USA) solution containing 0.01% H_2_O_2_, rinsed with PBS, dehydrated in alcohol, cleared in xylene, and cover-slipped with Entellan medium.

### Bromodeoxyuridine (BrdU)/Iba1 double immunofluorescence staining

Microglial proliferation in the brain after tMCAO challenge was determined by double immunofluorescence staining for BrdU and Iba1 at 3 days after tMCAO. BrdU (50 mg/kg, *i*.*p*., Sigma-Aldrich St. Louis, MO, USA) was given twice a day at 12 h intervals for 2 days after tMCAO. Brain sections were fixed with 4% PFA, exposed to 2 N HCl at 37 °C for DNA denaturation, and neutralized with borate buffer (0.1 M, pH 8.5). They were then blocked with 1% FBS in 0.3% Triton X-100 and labeled with primary antibodies against BrdU (1:200, Abcam, Cambridge, UK) and Iba1 (1:500) at 4 °C overnight. Sections were further labeled with secondary antibodies conjugated with Cy3 (1:1000, Jackson ImmunoResearch) and AF488 (1:1000, Invitrogen) and cover-slipped with VECTASHIELD^®^ mounting media (Vector Laboratories, Burlingame, CA, USA).

### NF-κB/Iba1 double immunohistochemistry staining

NF-κB signaling in activated microglia following tMCAO challenge was determined by NF-κB/Iba1 double immunohistochemistry staining as described previously^11^. Briefly, brain sections were fixed with 4% PFA, rinsed with PBS, and treated with Tris-EDTA solution at 100 °C for 30 min for antigen retrieval. Sections were blocked with 1% FBS in 0.3% Triton X-100 and incubated with rabbit NF-κB p65 (1:100, Santa Cruz, USA) antibody at 4 °C overnight. Sections were then incubated with biotinylated secondary antibody (1:200) at room temperature followed by incubating with ABC solution. Brain sections were then stained with DAB and washed with water. Stained sections with DAB were further labeled with a primary antibody against Iba1 (1:500) at 4 °C overnight. Sections were then incubated with a secondary antibody conjugated with Cy3 (1:1000) and mounted with VECTASHIELD^®^ mounting media.

### Image preparation and quantification

Brain images were obtained using a bright-field and fluorescence microscope equipped with a DP72 camera (BX53T, Olympus, Japan). Representative images were prepared using Adobe Photoshop Elements 8. For quantification, three images taken from different areas of each region were used for each mouse brain. The number of immunopositive cells were manually counted. The average number of cells is expressed in number of cells per unit area.

### Culture of BV2 microglia cell line and treatment

Murine BV2 microglial cells were cultivated in Dulbecco’s modified Eagle’s medium (DMEM) supplemented with 10% FBS, penicillin, and streptomycin. Cells were seeded onto 6-well plates at a density 2 × 10^5^ cells/well. To induce M1 polarization, BV2 cells were exposed to LPS (*Escherichia coli* serotype 026:B6, Sigma-Aldrich, St. Louis, MO, USA) for 24 h. JTE013 (2 µM) or vehicle (0.1% DMSO in DMEM) was added to cells at 30 min prior to LPS exposure. Alternatively, BV2 cells were transiently transfected with S1P_2_ siRNA or non-target control siRNA (NTC siRNA) as described previously^24^. Forty-eight hours later, cells were exposed to LPS.

### Western blot

Ipsilateral brain hemispheres were harvested at 24 h after tMCAO challenge and homogenized with neuronal protein extraction reagent. Protein samples from BV2 microglial cells were obtained at 1 h after LPS stimulation. Obtained proteins were separated through 10% SDS- PAGE, transferred to PVDF membrane, and blocked with 5% skim milk. These membranes were incubated with primary antibodies against rabbit phosphorylated forms of MAPKs (pERK1/2, pp38, and pJNK; Cell Signaling, 1:1000), total forms of MAPKs (ERK1/2, p38, and JNK; Cell Signaling, 1:1000), and β-actin (Sigma Aldrich, 1:5000) at 4 °C overnight followed by incubation with respective secondary antibodies (Jackson ImmunoResearch, 1:10000) at room temperature for 2 h. Protein bands were visualized with enhanced chemiluminescence solution. Densitometric analysis was carried out using Image J software (National Institute of Mental Health, Bethesda, MD) and normalized with β-actin.

### Quantitative real-time PCR (qRT-PCR) analysis

Total RNA was extracted from BV2 microglial cells or ipsilateral brain hemispheres using RNAiso plus (Takara, Kusatsu, Japan). For brain sampling, mice were perfused with autoclaved PBS and their brains were removed. Total RNA (1 µg) was used to generate cDNA by reverse transcription using All-in-One First-Strand cDNA Synthesis SuperMix (TransGen Biotech, Haidian, China). Then mRNA expression levels of M1 and M2 polarization markers were determined using StepOnePlus^TM^ qRT-PCR system (Applied Biosystems, Foster city, CA, USA) with FG Power SYBR Green PCR master mix (Life Technologies, Carlsbad, CA, USA) and specific primer sets (Supplementary Table 1). Expression levels of target genes were quantified using the 2^−ΔΔCT^ method relative to β-actin.

### Statistical analysis

All data are presented as mean ± standard deviation (SD). Statistical analysis was carried out using Mann-Whitney test for comparisons between two groups and one-way analysis of variance (ANOVA) followed by Newman-Keuls *post hoc* test for multiple comparisons using GraphPad Prism 5 (GraphPad Software Inc., La Jolla, CA, USA). Statistical significance was considered at *p* < 0.05.

## Supplementary information

Supplementary Information

## Data Availability

The data generated and analyzed as a part of this study are included within this article (as well as supplementary information).
