# Supplementary material for: S1P2 contributes to microglial activation and M1 polarization following cerebral ischemia through ERK1/2 and JNK
Source: Sci Rep. 2019 Aug 20;9:12106. doi: 10.1038/s41598-019-48609-z (PMC6702157; doi:10.1038/s41598-019-48609-z)
Supplement: Supplementary file 1 — Supplementary Information [file 41598_2019_48609_MOESM1_ESM.docx]

**S1P_2_ contributes to microglial activation and M1 polarization following cerebral ischemia through ERK1/2 and JNK**

Arjun Sapkota, Bhakta Prasad Gaire, Min-Gu Kang and Ji Woong Choi^*^

College of Pharmacy, Gachon University, 191 Hambakmoero, Yeonsu-gu, Incheon 21936, Republic of Korea

Email addresses of the authors:

Arjun Sapkota: [sapkotaa07@gmail.com](mailto:sapkotaa07@gmail.com); Bhakta Prasad Gaire: [samarpanbp@gmail.com](mailto:samarpanbp@gmail.com);

Min-Gu Kang: [eytro86@gmail.com](mailto:eytro86@gmail.com); Ji Woong Choi: [pharmchoi@gachon.ac.kr](mailto:pharmchoi@gachon.ac.kr)

^*^Corresponding author:

Ji Woong Choi, Ph.D. Laboratory of Neuropharmacology, College of Pharmacy, Gachon University, Yeonsu-gu, Incheon 406-799, Republic of Korea; Tel: +82-32-820-4955; Fax: +82-32-820-4829; E-mail: [pharmchoi@gachon.ac.kr](mailto:pharmchoi@gachon.ac.kr)

**Supplementary Information:**
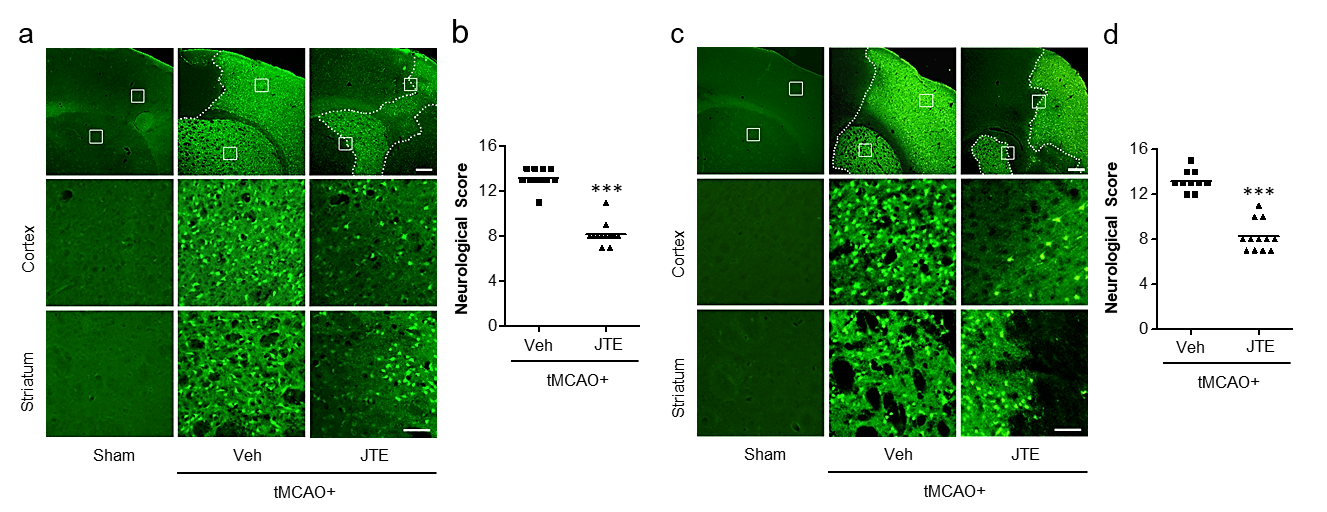


**Supplementary Figure 1.** **Suppressing S1P_2_ activity reduces neural cell death and improves neurological functions of tMCAO-challenged mice.** Effects of JTE013 (JTE) on neuronal cell death were observed by FJB staining. **a, c** Representative photographs of FJB staining in post-ischemia damage in cortex and striatum regions at 1 day (**a**) and 3 days (**c**) after tMCAO challenge. Diagram box in the upper panel shows the cerebral area where images in middle and bottom panels are obtained. Dotted lines separate periischemic and ischemic core regions. Scale bars, 200 µm (top) and 50 µm (middle and bottom). (**b, d**) Neurological score for neurological function at 1 day (**b**) and 3 days (**d**) after tMCAO challenge. n = 12 per group. ^***^*p* < 0.001 vs. vehicle-treated group by Mann-Whitney test.

Supplementary Table 1. Primer sets used for qRT-PCR analysis

| **Gene** | **Direction** | **Sequence** | Gene Accession # |
| --- | --- | --- | --- |
| β-actin  (NM_007393) | Forward | 5'-AGCCTTCCTTCTTGGGTATG-3' | NM_007393 |
|  | Reverse | 5'-CTTCTGCATCCTGTCAGCAA-3' |  |
| TNF-α  (NM_013693) | Forward | 5'-CATCTTCTCAAAATTCGAGTGACAA-3' | NM_013693 |
|  | Reverse | 5'-TGGGAGTAGACAAGGTACAACCC-3' |  |
| IL-6  (NM_031168) | Forward | 5'-GAGGATACCACTCCCAACAGACC-3' | NM_031168 |
|  | Reverse | 5'-AAGTGCATCATCGTTGTTCATACA-3' |  |
| IL-1β  (NM_008361) | Forward | 5'-CAACCAACAAGTGATATTCTCCATG-3' | NM_008361 |
|  | Reverse | 5'-GATCCACACTCTCCAGCTGCA-3' |  |
| CD11b | Forward | 5'-CCCCAATTACGTAGCGAATG-3' | NM_001082960 |
|  | Reverse | 5'-TGCTGCGAAGATCCTAGTTG-3' |  |
| CD16 | Forward | 5ˈ-TATGGCACCTTAGCGTGATG-3ˈ | NM_010188 |
|  | Reverse | 5ˈ-CGACCCTGTAGATCTGGGAG-3ˈ |  |
| CD32 | Forward | 5ˈ-CTCGAGTTTGACCACAGCCT-3ˈ | NM_001077189 |
|  | Reverse | 5ˈ-TGTTCTCACGGACTTTGTGC-3ˈ |  |
| CD86 | Forward | 5ˈ-TCTCCACGGAAACAGCATCT-3ˈ | NM_019388 |
|  | Reverse | 5ˈ-CTTACGGAAGCACCCATGAT-3ˈ |  |
| Arg1 | Forward | 5ˈ-TTTTTCCAGCAGACCAGCTT-3ˈ | NM_007482 |
|  | Reverse | 5ˈ-AGAGATTATCGGAGCGCCTT-3ˈ |  |
| CCL-22 | Forward | 5ˈ-TGGAGTAGCTTCTTCACCCA-3ˈ | NM_009137 |
|  | Reverse | 5ˈ-TCTGGACCTCAAAATCCTGC-3ˈ |  |
| CD206 | Forward | 5ˈ-GTGGATTGTCTTGTGGAGCA-3ˈ | NM_008625 |
|  | Reverse | 5ˈ-TTGTGGTGAGCTGAAAGGTG-3ˈ |  |
| IL-10 | Forward | 5ˈ-TGGCCTTGTAGACACCTTGG-3ˈ | NM_010548 |
|  | Reverse | 5ˈ-AGCTGAAGACCCTCAGGATG-3ˈ |  |
| TGF-β1 | Forward | 5ˈ-CAACCCAGGTCCTTCCTAAA-3ˈ | NM_011577 |
|  | Reverse | 5ˈ-GGAGAGCCCTGGATACCAAC-3ˈ |  |
| Ym1 | Forward | 5ˈ-TTTCTCCAGTGTAGCCATCCTT-3ˈ | NM_009892 |
|  | Reverse | 5ˈ-AGGAGCAGGAATCATTGACG-3ˈ |  |

**Supplemental Western blots:**

**
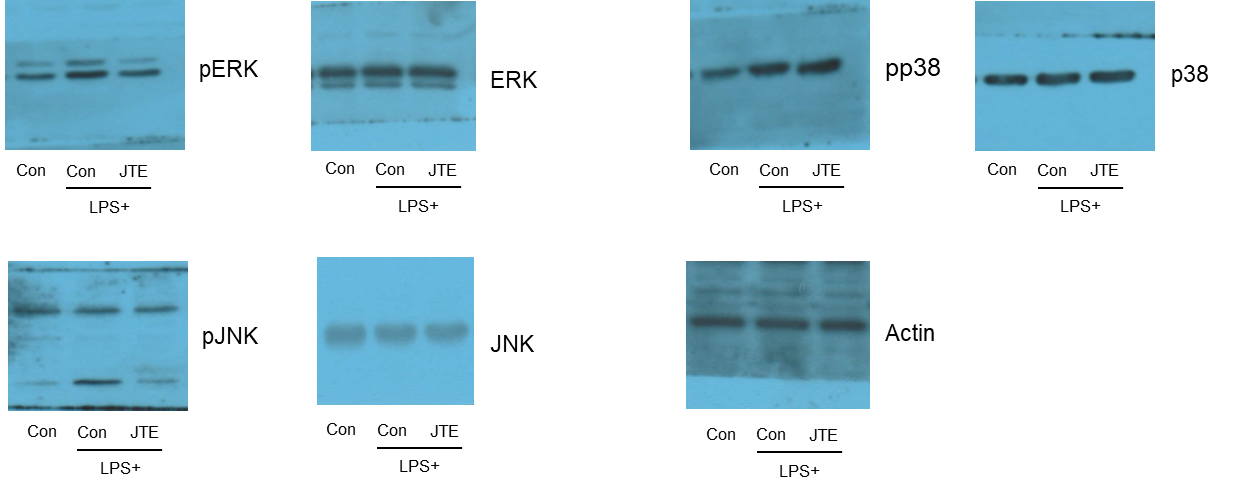
**


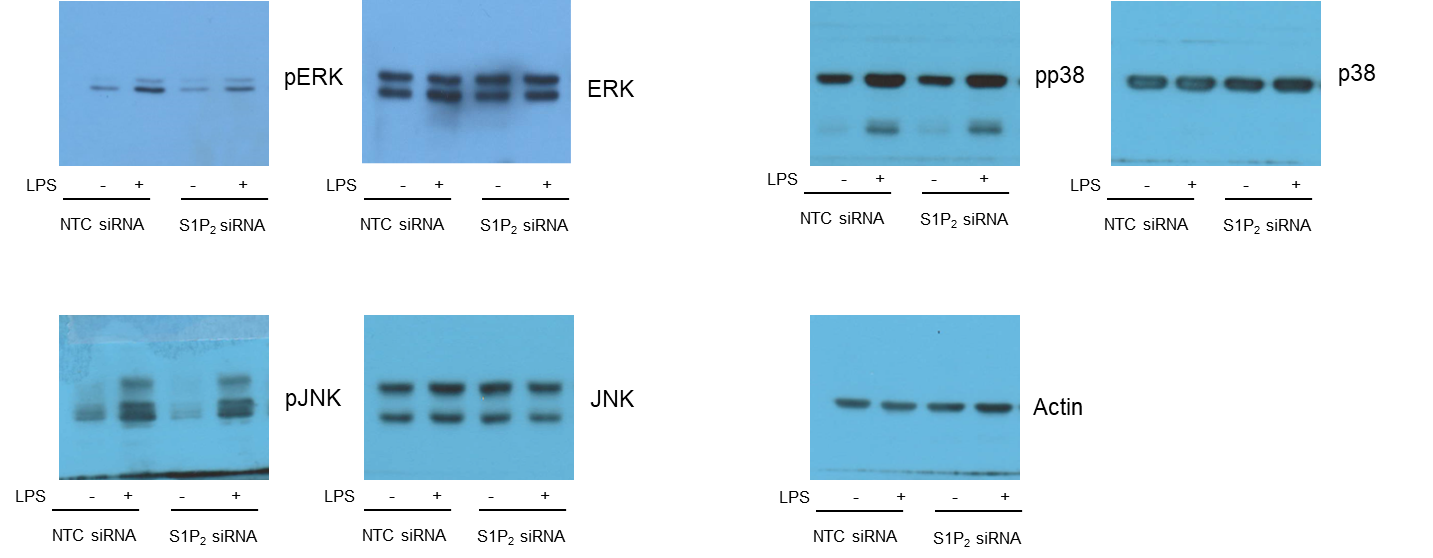


Original Western blots related to Fig. 7.


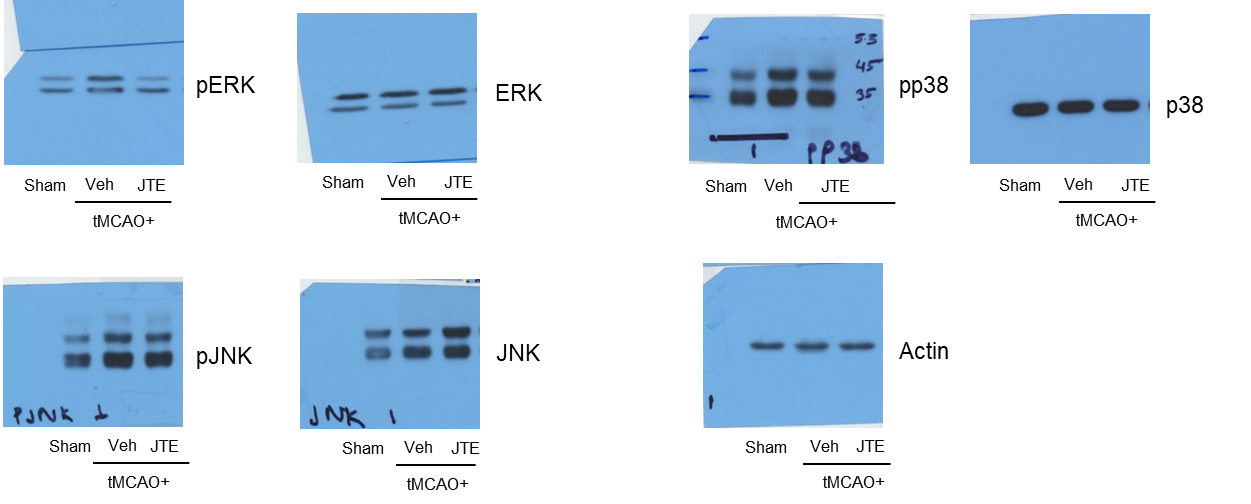


Original Western blots related to Fig. 8.
